# Diet Assessment Based on Rumen Contents: A Comparison between DNA Metabarcoding and Macroscopy

**DOI:** 10.1371/journal.pone.0157977

**Published:** 2016-06-20

**Authors:** Ruth V. Nichols, Mikael Åkesson, Petter Kjellander

**Affiliations:** 1 Grimsö Wildlife Research Station, Department of Ecology, Swedish University of Agricultural Sciences, Riddarhyttan, Sweden; 2 Molecular Ecology Group, Department of Wildlife, Fish and Environmental Studies, Swedish University of Agricultural Sciences, Skogsmarksgränd, Umeå, Sweden; 3 Department of Ecology and Evolutionary Biology, University of California Santa Cruz, Santa Cruz, California, United States of America; University of Guelph, CANADA

## Abstract

Dietary choices are central to our understanding of ecology and evolution. Still, many aspects of food choice have been hampered by time consuming procedures and methodological problems. Faster and cheaper methods, such as DNA metabarcoding, have therefore been widely adopted. However, there is still very little empirical support that this new method is better and more accurate compared to the classic methods. Here, we compare DNA metabarcoding to macroscopic identifications of rumen contents in two species of wild free-ranging ungulates: roe deer and fallow deer. We found that the methods were comparable, but they did not completely overlap. Sometimes the DNA method failed to identify food items that were found macroscopically, and the opposite was also true. However, the total number of taxa identified increased using DNA compared to the macroscopic analysis. Moreover, the taxonomic precision of metabarcoding was substantially higher, with on average 90% of DNA-sequences being identified to genus or species level compared to 75% of plant fragments using macroscopy. In niche overlap analyses, presence/absence data showed that both methods came to very similar conclusions. When using the sequence count data and macroscopic weight, niche overlap was lower than when using presence-absence data yet tended to increase when using DNA compared to macroscopy. Nevertheless, the significant positive correlation between macroscopic quantity and number of DNA sequences counted from the same plant group give support for the use of metabarcoding to quantify plants in the rumen. This study thus shows that there is much to be gained by using metabarcoding to quantitatively assess diet composition compared to macroscopic analysis, including higher taxonomic precision, sensitivity and cost efficiency.

## Introduction

Many central questions in ecology focus on the use and selection of food by individuals, populations or species. Particularly, in large herbivores the applications of such knowledge of the diet is wide ranging. For example, management related questions and the consequences for commercially valuable crops and trees are dependent on knowledge about diets [1]. In population dynamics, diet is an important driver of intraspecific competition [2]. In behavioral ecology, diet is used as a correlate to fitness [3]. In conservation ecology, diet is used both in terms of effects on the ecosystem and in terms of preserving threatened species [4]. Most studies of wild ungulate diets use traditional techniques such as macroscopic ruminal, or histological fecal analysis. However these techniques are hampered with inherent methodological problems [5] that involve inaccurate determination of fragmented plant material or simply missing the very small fragments [6]. This may result in a bias due to differential digestibility, i.e. when some food items are digested more rapidly than other food items. Thus quantitative comparisons between different foods are only approximate indications of their relative importance [6].

DNA-based diet assessment, e.g. DNA metabarcoding, has rapidly increased in popularity over the past few years as a way to assess the diets of animals [7, 8]. Successful DNA barcoding, i.e. the identification of taxa using DNA sequences [9], relies on finding genetic regions were different taxa are distinguishable and at the same time are flanked by highly conserved DNA for primer annealing. Metabarcoding uses the same principle, yet it uses Next Generation Sequencing technology to barcode multiple sequences on several samples simultaneously. This method has been shown to be both time efficient and give precise identification of amplified DNA sequences [4, 8, 10].

There are now studies presenting results in favor of using DNA metabarcoding instead of microhistology or macroscopic analyses [10]. The empirically tested advantages of metabarcoding include higher sensitivity, higher taxonomic detail and higher cost efficiency [11]. However, the few studies that have investigated such differences are specific to a few study systems, such as diatoms [12], skinks [13] and the diet of voles [10]. While these studies are important, it is difficult to draw general conclusion across different study systems and taxa. Moreover, the current literature on herbivorous metabarcoding focuses heavily on using DNA from faeces [4, 10, 14–19]. Even though faeces have the advantage of being non-invasive, many studies on large herbivores use rumen samples instead of faeces, in order to gain higher taxonomic precision and lower bias due to differences in digestibility between different plant species and parts of plants [20, 21].

It is vital to understand how alternative methods compare to each other. However, without controlled experimental studies, it is difficult to know the strengths and weaknesses of the methods and thereby their relative suitability for different purposes. An important aspect of using any method to study diet is to question whether or not the proportion of food items identified accurately reflects the relative proportions of food items consumed. Empirical studies examining this are, thus far, few and conflicting. One experimental study found a positive correlation between the proportions of plants fed to sheep and the proportions of DNA sequences recovered [22]. However this study examined low complexity diets (only two plant species were used). In other studies on mixed diets, little correlation was found between biomass consumed and number of DNA sequences retrieved from faeces [23, 24]. Controlled experiments where the amount of food consumed is known can be impractical in free-ranging or wild animals with heavily mixed diets. Thus, it is more practical to compare the results of two methods for examining diets after the animals have eaten. Such studies have found positive relationships between the proportions of different growth forms (i.e. forbs and graminoids) found using stable isotopes and DNA metabarcoding [25, 26]

This study resolves a gap in the literature regarding the comparability of methods, with particular reference to ungulates. Here, we use both macroscopy and DNA metabarcoding on the same rumen samples in two species of ungulates and we assess the comparability of the two methods. The native roe deer (*Capreolus capreolus*) is considered as a browser whereas the introduced fallow deer (*Cervus dama*) is known as more of a mixed feeder [27]. As a non-native species, fallow deer is expected to have a wider resource overlap with the sympatric native species in the area where it is introduced than is observed among the native species in the same area [28, 29] Yet we expect them to occupy different niches, based on past work [30]. Here we calculated niche overlap using both the DNA metabarcoding and macroscopy data in order to compare the performance of the two methods when answering an ecological question.

## Materials and Methods

### Ethics statement

Ethical approval was granted for this research project by the Gothenburg Board for Laboratory Animals (Dnr: 405–2008) and we observe the ASAB/ABS Guidelines for the Use of Animals in Research.

### Study area

This study was performed at the Koberg Estate (58˚ N, 12˚ E) in south-western Sweden (Västra Götaland County). The study area (54.35 km^2^) is mostly covered by deciduous, coniferous or mixed types of forest (79%), and the remaining area constitutes arable land and pastures (16%), mires and marshes (2%), lakes, ponds, parks and properties around houses (3%). The two most common habitat types are coniferous forest > 15 m high (29%) and coniferous forest 5–15 m high (15%); [31]. The open landscape at Koberg today, consisting of arable land and pastures, is to a large extent cultivated in order to improve wildlife habitats, and supplementary food is also given during winter. While roe deer is a native species to the area, free ranging fallow deer has been present since the release of a few animals (approximately 20) from an enclosure in the end of the 1920’s (Count Niclas Silfverschiöld unpubl. data). Other ungulates occurring in the area are moose (*Alces alces*) and wild boar (*Sus scrofa*) and low numbers of red deer (*Cervus elaphus*) and mouflon (*Ovis orientalis*). Controlled hunting of fallow deer is performed each fall (September-February, with a pause during the rut) and potential predators present in the area are the red fox (*Vulpes vulpes*) and occasional visits of lynx (*Lynx lynx*) and wolf (*Canis lupus*).

### Macroscopic identification of rumen content

Rumen content was collected as a part of a larger study on ungulate diet from free ranging fallow deer and roe deer killed during regular hunting and also during other times of the year, from road kills or shot by trained professional hunters with the ethical approval from the Gothenburg Board for Laboratory Animals (Dnr: 405–2008) and a permit from the Swedish Environmental Protection Agency (Dnr: NV-08702-12). In this paper we only use data from animals killed in July and August (summer) 2007 and 2011. After removing the total intestine from the killed animal the rumen was opened and the contents mixed, in order to decrease effects of a structured plant representation. A sample of 1 liter per rumen was taken representing 10–30% of the total rumen content [32] and stored at -20 ˚C. Macroscopic identification was initiated by thawing a rumen sample and taking out 250 mL that was washed under water and over a sieve with a 4 mm mesh size. The fragments retained by the 4 mm sieve were determined to the lowest taxonomic level possible using Mossberg and Stenberg [33] and our own reference collections of plants. After determination, the material was dried at 70°C for ≥72h and each identified taxa was weighed to nearest 0.01 g. This methodology is used in a study of moose and roe deer rumen content described by Cederlund et al. [20].

### DNA Laboratory work

All samples (10 fallow deer and 9 roe deer) were prepared by homogenizing 10–50 ml of mixed rumen content, using mortar, pestle and liquid nitrogen. Whole DNA was extracted from a subsample of the homogenized rumen content (100 mg wet weight) with the DNeasy plant Mini kit (QIAGEN Inc.) following the manufacturer’s instructions. The final elution volume was 200 μL. All extractions contained negative controls to account for possible contaminations.

An Ion Torrent amplicon library was prepared using the fusion PCR method. Fusion primers were prepared based on the target sequence of primers *g* and *h* described in [34], to enable unidirectional sequencing of the *trnL* (UAA) intron located in the chloroplast DNA. Both primers were fitted with an adaptor sequence at the 5’-end and the *g* primers with a sample specific barcode sequence, in accordance with the manufacturer’s instructions for Ion Amplicon Preparation. DNA amplifications were carried out in a final volume of 50 μL, including 45μL Platinum® PCR SuperMix High Fidelity (Invitrogen), 1 μL template DNA and 200 nM of each fusion primer. The PCR reaction involved 2 min incubation at 94°C, followed by 35 cycles of 30 s at 94°C, 30 s at 55°C and 30 s at 68°C, and ended with 10 min incubation at 68°C. All PCR runs included a negative control, without sample material. All PCR products were purified using QIAquick PCR Purification Kit (Qiagen, Inc.) in accordance with the manufacturer’s instructions. All PCR-products, including negative controls, were verified for successful amplification and possible contamination using agarose gel electrophoresis. All samples were then pooled and sequenced on a single 316 chip using an Ion Torrent PGM^TM^ system (Life Technologies, Inc.).

### Reference library

We compiled a reference library based on four separate vegetation surveys done in the Koberg study area during both winter (January-March) and summer (July-August) in 2007 and 2010. Each survey covered the study area by a 150 x 150 m grid system and the positions of >2000 sampling plots each time were programmed into handheld GPS units (Garmin 60CSx and Garmin 12XL), thus generating >8000 unique sampling plots. At each plot the vegetation, excluding mosses, ferns and lichens, within a 25 x 25 cm square up to a height of two meters were identified to the species level. Using this data as a species list, we extracted sequences from the *arctic trnL* reference library [35], the *boreal* and *embl* reference libraries [22], and searched for any remaining sequences on Genbank. We compiled these sequences into our own regional reference library. Most of the taxa found using the vegetation survey were able to be included in the reference library. Only 23 out of 263 taxa were not able to be included. The final library includes taxa at the finest resolution possible. For some taxa this means that they were at the genus level (32%) or the family level (7%), while most were at the species level (61%).

### Bioinformatics

After sequencing, the primer tags were used to sort sequences by sample. One file per sample was made, containing all sequences specific to a sample. We used the OBITools package (http://metabarcoding.org/obitools) to group unique sequences and filter out any errors due to PCR. The filtering step was strict and used an R (ratio) value of 1. We ignored sequences with DNA sequence counts less than 15. Cut-off values from other studies can range from 3–1000 [10, 22]. We chose a cut-off value of 15 in order to keep rare species in our data set but also to limit the number of potentially spurious results. To match sequences to our reference library we used the Usearch algorithm [36] with matches needing to have ≥ 99% identity. Anything that did not match the reference library, we identified with ≥ 99% identity by using BLAST in NCBI’s nucleotide collection (nr/nt).

### Data analysis

The identifications via the different methods had different taxonomic precision, thus in order to compare the methods, we separated the data into different sets using different taxonomic levels (i.e. Family, Genus and Species). In order to investigate if there was variation in detection and quantification among different growth forms, we added a fourth ‘Functional’ grouping consisting of deciduous trees, shrubs, conifers, herbs, graminoids, crops, ferns, mosses, lichens, and fungi.

In order to get a measure of reproducibility between the two methods, we used a similarity measure. We calculated the Jaccard coefficient of similarity (S_J_) within individual samples by dividing the number of taxa in common by the total number of taxa identified by both methods within a sample. We then calculated mean coefficients of similarity.

It is a common question in the literature as to whether or not the number of sequences retrieved reflects the actual amount of material eaten [23]. The proportion of material (dry weight) found macroscopically may roughly reflect the proportion of material consumed in the diet [6, 20, 37]. We thus investigated whether or not the proportion of DNA sequences correlated to the proportion weighed macroscopically using the data where we had quantities above zero for both methods. We tested the correlation between proportion of DNA sequences and proportion of macroscopic weight using Spearman’s ρ. We used the logarithm of the proportional values to generate a symmetric frequency distribution [38].

To test if DNA detection rate depended on the plant specific weight identified from macroscopic analysis, we used generalized linear mixed models (GLMMs) in R (v2.15.2), with the lmer-function in lme4 assuming binomial distribution, logit link-function and Laplace maximum likelihood. The non-independence of detection rate within samples was accounted for by treating sample ID as a random effect. To increase logit linearity we used the logarithm of the sample weight. This analysis was conducted on several phylogenetic levels, including family, genus and species, and on functional groups. Prior to analysis at each phylogenetic level we omitted observations of plants with sequences that were non-unique in relation to other plants in the reference library. On family and functional group level all sequences were distinguishable, while seven genera (*Avena*, *Matricaria*, *Polypodium*, *Ribes*, *Sorbus*, *Taraxacum* and *Urtica*) and 12 species (*Acer platanoides*, *Achillea millefolium*, *Achillea ptarmica*, *Avena sativa*, *Matricaria perforata*, *Persicaria lapathifolia*, *Ranunculus repens*, *Rumex acetosella*, *Sorbus aucuparia*, *Stellaria graminea*, *Trifolium medium* and *Trifolium repens*) were not distinguishable and thus omitted prior to GLMM analyses. An alternative to omitting indistinguishable taxa would be to combine them to a higher distinguishable taxonomic level. This was however complicated to do when the higher taxonomic level also included taxa that indeed were distinguishable. Thus, we managed to assign 9 of 12 indistinguishable species and 1 of 7 genera to a higher taxonomic level. The results from this analysis did not change the general conclusions of the study (result not included).

A positive association between DNA-detection probability and plant specific weight could be explained by low-quantity food-items being missed when taking a sample of the rumen content for DNA analysis. It could also be explained by a weight dependent effect when determining species/genus macroscopically. To investigate this further we singled out ten species (*Calluna vulgaris*, *Gymnocarpium dryopteris*, *Picea abies*, *Pinus sylvestris*, *Plantago major*, *Quercus robur*, *Rubus idaeus*, *Urtica dioica*, *Vaccinium myrtillus* and *Vaccinium vitis-idaea*) that were particularly easy to determine macroscopically and tested if DNA detection rate depended on the plant specific weight.

To see how the different methods performed when analysing dietary niches, we calculated niche overlap between fallow deer and roe deer using the DNA metabarcoding method and the macroscopic method. We used the methodology and R scripts found in Geange et al. [39]. In summary, Geange et al. [39] provide statistical tests for collapsing multiple data types representing multiple niche axes such that total niche overlap can be calculated between different species. Overlap is calculated using permutations which then provide a p-value to test the significance of the overlap. We ran these analyses using 1) raw sequence count/macroscopic weight data, and 2) binary (presence/absence) data, and we did these two analyses on all data sets (functional, family, genus, species). Each plant functional group or taxa represented a different niche axis to which overlap was calculated. The final niche overlap estimates incorporates all niche axes (e.g. all food items). We used permutation analyses with 1000 replications to obtain p-values that indicate whether or not roe deer and fallow deer occupy significantly different niche spaces. When running the permutation analysis for the binary data, we removed vectors that were non-variable.

## Results

### Sequence filtering

Sequencing yielded a total of 2,855,866 reads, while the number of reads per sample varied from 28,248 to 890,050 (Mean = 150,309 ± 181,094 S.D.). After filtering, 834,001 were left. From these remaining sequences, a total of 7,435 sequences were unidentified and 826,566 sequences were identified at the family, genus and/or species taxonomic levels. In total, 86% of the sequences were identified using the reference library whereas 14% of the sequences were identified using BLAST.

### Taxonomic precision and similarity between methods

**Fig 1:**
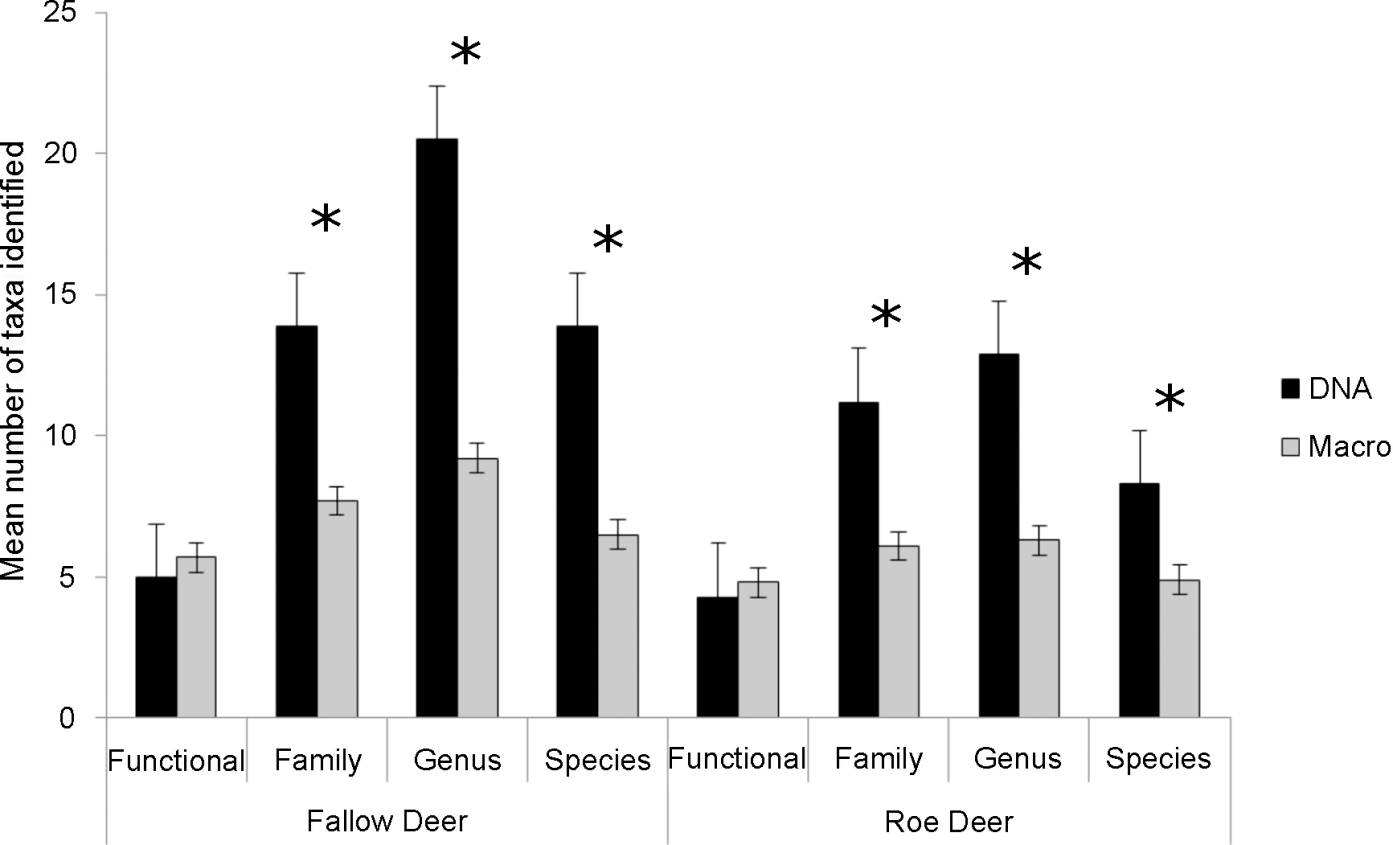
Rumen content in fallow deer and roe deer using two detection methods. Mean number of taxa identified in the rumen of fallow deer and roe deer, using DNA-based metabarcoding and macroscopy. Significant differences are indicated with * p < 0.01. Error bars represent standard error.

**Fig 2:**
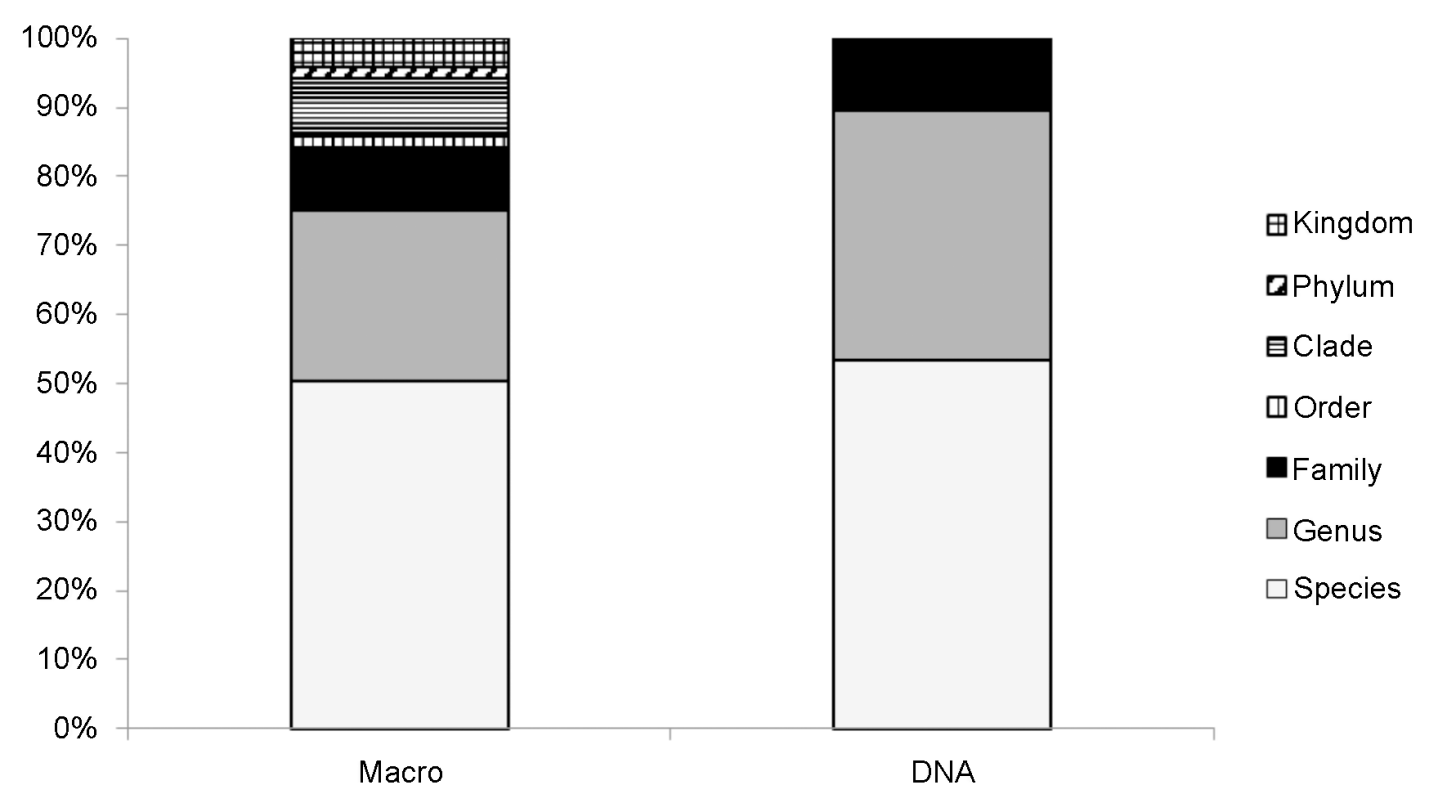
Taxonomic precision of ungulate rumen content based on two different methodologies i.e. macroscopic identification (Macro) and DNA metabarcoding (DNA). Proportions are based on the average number of identified objects across samples down to the lowest phylogenetic level. Category order includes unspecified graminoids, clade includes unspecified deciduous browse and herbs, phylum includes unspecified mosses and kingdom includes unspecified fungi.

The mean number of identified taxa was greater when using DNA than when using the macroscopic method for all groupings except for the functional grouping (Fig 1). Taxonomic precision increased using DNA, where on average 90% of sequences could be identified to the genus or species level using DNA, compared to 75% of plant fragments using macroscopy (Fig 2). Similarities between methods decreased with lowered taxonomic level/classification (i.e. the lowest taxonomic level being species). At the level of the functional group, mean *S*_*J*_ ± S.D. similarity within samples was 0.65 ± 0.16; at the family level it was 0.38 ± 0.13; at the genus level it was 0.23 ± 0.09; at the species level it was 0.21 ± 0.09).

**Fig 3:**
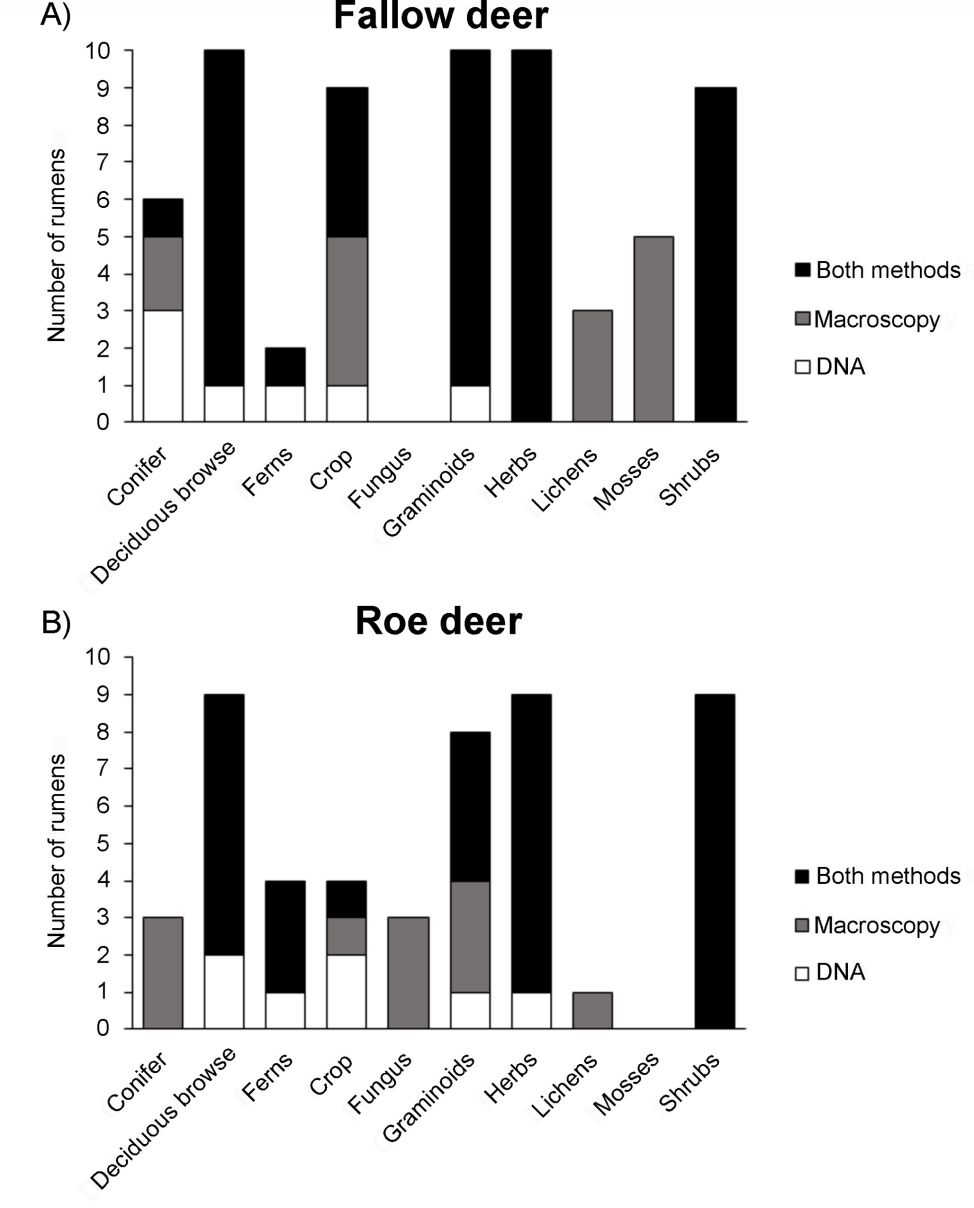
Representation of different functional groups in the rumen of fallow deer and using deer using two detection methods. The total number of rumen containing different functional groups determined by either macroscopic identification or DNA metabarcoding or by both methods in A) fallow deer (N = 10) and B) roe deer (N = 9) from Southwestern Sweden.

At the functional group level, all ruminal observations of deciduous browse (n = 19), ferns (n = 6), herbs (n = 19) and shrubs (n = 18) were detected either by both methods or solely by the DNA-based method, and the proportion of observations missed by macroscopic analysis was 16% of the deciduous browse, 33% of the fern, 5% of the herb and 0% of the shrub observations (Fig 3). Conifers, crop plants and graminoids were detected to a varying degree by both or either one of the two methods. The observations made solely macroscopically were 55% observations of conifers (n = 9), 38% of the crop plants (n = 13), and 17% of the graminoids (n = 18).The observations made solely by DNA were 33% of the conifers, 23% of the crop plants and 11% of the graminoids. For fungi (n = 3), lichens (n = 4) and mosses (n = 5) all observations were made solely macroscopically.

At the family level 137 of 293 (47%) observations were made solely by DNA, while 53 (18%) were made solely macroscopically and the result differed only slightly between roe deer and fallow deer (S1 Table). At the genus level 231 of 413 (56%) observations were made solely by DNA, while 92 (22%) were made solely macroscopically (S2 Table). At the species level 158 of 285 (55%) observations were made solely by DNA, while 71 (25%) were made solely macroscopically (S3 Table).

### Quantity relationship

**Fig 4:**
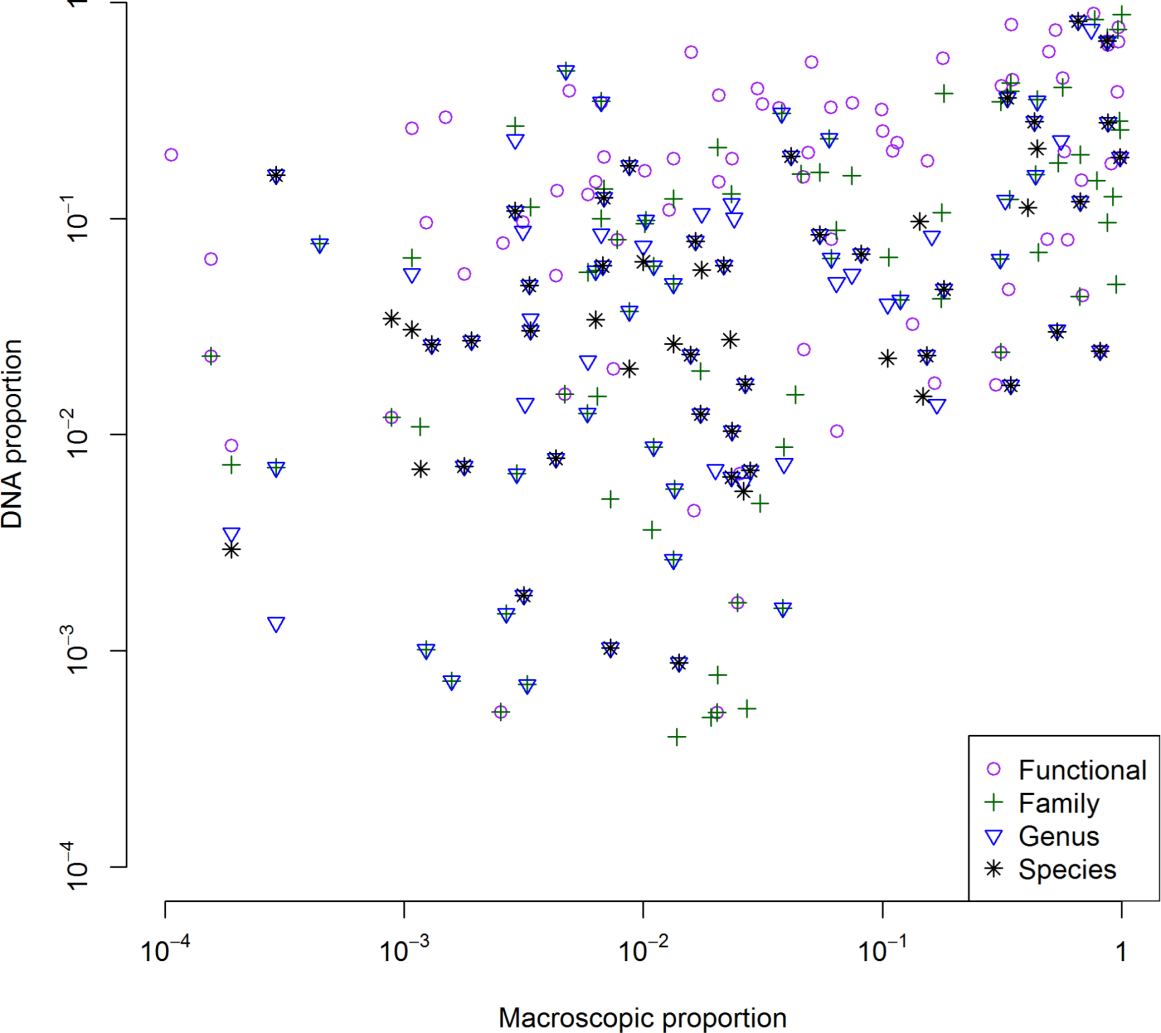
Correlations between the proportional quantity of different food items from DNA metabarcoding and macroscopic identification. The plotted association between the two methods is based on four different levels, including the ‘functional group’ level in purple open circles, the family level in green plus signs, the genus level in blue triangles and the species level in black asterisks.

We found that the log-proportion of DNA reads and log-proportion of macroscopic weight were significantly correlated (Fig 4). At the functional group level Spearman’s ρ = 0.39 (P = 0.0004); at the family level Spearman’s ρ = 0.52 (P < 0.0001); at the genus level Spearman’s ρ = 0.42 (P < 0.0001); and at the species level Spearman ρ = 0.39 (P = 0.0043).

### Detection probability

DNA-detection probability showed clear indications of being dependent on plant specific weight on all phylogenetic levels (Table 1A) We did not find any difference in DNA detection probability between the two ungulate species (Family level: n = 127, z = 1.11, P = 0.3; Genus level: n = 126, z = 0.98, P = 0.3; Species level: n = 82, z = 1.27, P< 0.2; Functional group level, n = 100, z = 0.00, P = 1.0). Moreover, we did not detect any significant effect of sequence length on the probability of DNA detection at the species level (n = 82, z = 0.98, P = 0.33). To further investigate if the weight dependent DNA detection probability was due to errors in macroscopically determining some species in low quantity, we analyzed the data on a subsample with only ten of the most easily macroscopically determined plant species. Also, with this reduced data set we found that DNA detection probability depended on plant weight (β = 0.89 ± 0.42 S.E., n = 57, z = 2.12, P = 0.03), which further indicates that low-quantity food items were not represented (or represented in too low quantity) in the samples taken from the rumen content.

**Table 1:** Model parameter values explaining detection probability of food items in rumen at different taxonomic levels using A) metabarcoding and B) macroscopic analysis. Sample-ID (rumen) was included as a random effect in all models.

				Intercept			Quantity		
Taxonomic level		n		Estimate ± SE	z	P	Estimate ± SE	z	P
*A) DNA detection probability (Quantity = log(Macroscopic weight))*									
Family	127		3.15 ± 0.69		4.60	< 0.001	1.17 ± 0.33	3.57	< 0.001
Genus	126		2.94 ± 0.61		4.80	< 0.001	1.28 ± 0.31	4.10	< 0.001
Species	82		2.45 ± 0.64		3.81	< 0.001	1.14 ± 0.34	3.38	< 0.001
Functional group	100		2.08 ± 0.45		4.65	< 0.001	0.77 ± 0.26	2.98	0.003
*B) Macroscopic detection probability(Quantity = log(DNA sequence count))*									
Family	240		-2.87 ± 0.50		-5.76	< 0.001	0.90 ± 0.17	5.32	< 0.001
Genus	320		-3.93 ± 0.52		-7.55	< 0.001	1.09 ± 0.18	6.17	< 0.001
Species	212		-4.66 ± 0.73		-6.36	< 0.001	1.34 ± 0.26	5.26	< 0.001
Functional group	89		-3.41 ± 1.35		-2.52	0.001	1.70 ± 0.50	3.37	< 0.001

Macroscopic-detection probability showed clear indications of being dependent on plant specific DNA count on all phylogenetic levels (Table 1B).

### Niche Overlap

**Fig 5:**
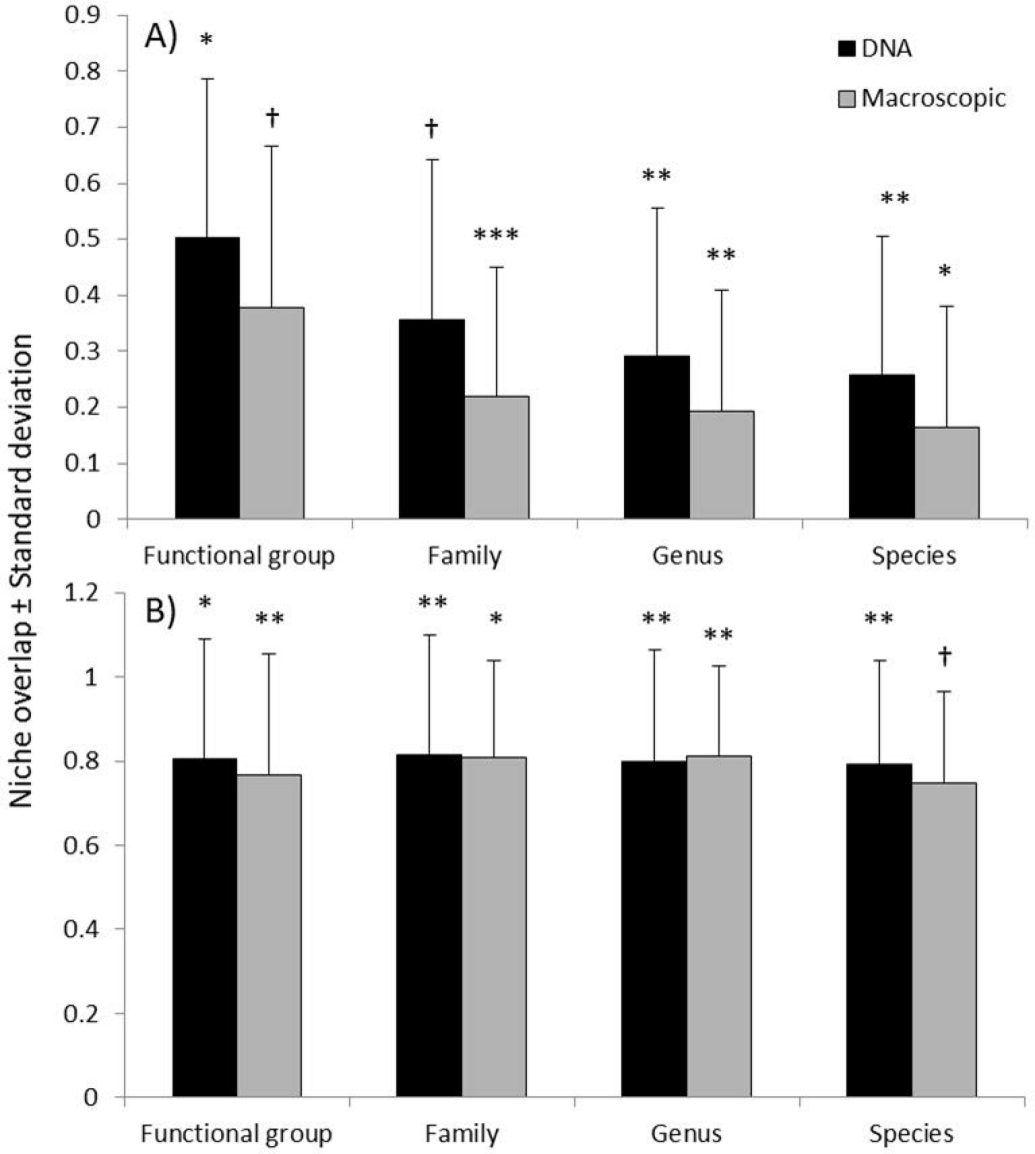
Niche overlap between fallow deer and roe deer using two different methodologies to determine rumen diet content. (A) Niche overlap based on quantitative data using dry weight (g) of macroscopically identified food items and DNA sequence count from metabarcoding. (B) Niche overlap based on binary data using on presence and absence of food plant species identified macroscopically or with DNA. Significant niche differences are indicated with *** p < 0.001, ** p < 0.01, * p <0.05 and † p < 0.1.

When using quantitative estimates of food items (i.e. food item weights from the macroscopic analysis and DNA sequence counts from the metabarcoding) the average niche overlap was estimated to 0.30 ± 0.10 (S.D.). The difference in dietary niches between fallow deer and roe deer was significantly different for all phylogenetic levels and detection methods (P < 0.05 in most cases) except for the functional group level using macroscopic detection (P < 0.1) and the family level using DNA-based detection (P < 0.1) (Fig 5). The niche overlap was on average 11% lower (across all phylogenetic levels) when using the macroscopic method compared to the DNA-method (Fig 5A). Niche overlap decreased with increasing taxonomic resolution. Using the DNA method the niche overlap changed from 0.50 ± 0.28 (S.D.) on functional group level to 0.26 ± 0.16 (S.D.) on species level. A similar pattern was seen with the macroscopic method where the niche overlap changed from 0.38 ± 0.29 (S.D.) on functional group level to 0.16 ± 0.22 (S.D.) on species level.

When using only the presence versus absence of food items in the rumen, niche overlap was on average 0.79 ± 0.02 (S.D.), thus higher than the niche overlap based on quantitative data (Fig 5B). The difference in dietary niches was significantly different for all phylogenetic levels and detection methods (P < 0.05 in most cases) except for the species level using macroscopic detection (P < 0.1). Moreover, with the use of presence versus absence of food items there were only very small differences in niche overlap between methods and taxonomic levels.

## Discussion

We used DNA metabarcoding to analyze diet composition based on rumen content from two large herbivores living in sympatry, roe deer and fallow deer, and compared it to a classical macroscopic method to determine food items. Although the DNA metabarcoding method proved to be both accurate and comparable there are differences between the two methods that ultimately may affect important ecological conclusions.

Overall we found a greater number of identified taxa when using DNA (Fig 1). This may reflect the fact that food material stays in the rumen for only a few hours before it is ruminated and later passed along to the stomach [40]. Thus, after rumination some plant species might become unidentifiable macroscopically, while they still are detectable using DNA. Thus, using DNA methods may also increase the window of time for detection of some food items. Similarity measures decreased when moving from high (functional) to low (species) taxonomic level. Partly this is expected, because there is more opportunity for variation between methods with lower taxonomic resolution, and partly it could be explained by the increased ability to determine plants to a lower taxonomic level with DNA than is possible macroscopically. The latter is reflected in the increased taxonomic precision we found when using DNA (Fig 2), which is also what other studies have found [10]. We saw differential rates of identification depending on functional group (Fig 3). For instance, fungi were never identified via DNA metabarcoding, but this is to be expected, because they do not contain chloroplast DNA. Lichens and mosses, on the other hand, do have chloroplasts, but they are often difficult to identify using the *g*/*h* primer pair used here [34]. To get a more comprehensive documentation of the food composition of herbivores, other primers could be used in tandem with the *g*/*h* primers to capture a large range of taxa. Additionally, we found that the detection rate from DNA clearly was lower for plants that were present in lower quantities in the rumen. This could indicate that the quantity of rumen content extracted for PCR was too low, even though the rumen content was thoroughly mixed before sample extraction. It is therefore advisable that a larger and more standardized quantity of rumen content is sampled prior to homogenization and DNA isolation. Moreover, the macroscopic detection also depended on the DNA sequence count, which is likely to be explained by the inability to determine small and fragmented food items macroscopically

In order to compare methods when answering an ecological question, we estimated dietary niche overlap between roe deer and fallow deer. In general, we found that using presence/absence data, the overlap did not differ much between methods nor between taxonomic groupings (Fig 5). On the other hand, when using quantitative data (sequence counts for DNA and weights for macroscopic analysis) niche overlap indices differed. Overall, niche overlap was higher when using DNA. At the functional level, the two species were found to occupy different niches using DNA, whereas they were not different when using macroscopic data. The reverse was true at the family level. At the genus and species levels, both DNA and macroscopic analysis showed that the two species occupied different niches. Interestingly, we found a clear pattern of decreasing niche overlap with increasing taxonomic resolution, indicating strong interspecific, perhaps competitive effects between the two sympatric large herbivores, roe and fallow deer. This has in fact been suggested previously for these two species, in a study based on radio tracking and habitat selection data [e.g. 41]. All together, these results indicate that past studies using presence/absence data from macroscopic analysis may be comparable to present studies using DNA. Still, without taking quantitative differences into account we are likely to overestimate the importance of less frequent food items and vice versa. It is therefore preferable to base measures of niche-overlap on quantitative data.

Even though it is preferable to calculate niche-overlap using quantitative data, available literature is conflicting as to the use of the number of DNA reads as a quantitative proxy for how much plant material has been consumed [24]. Several alternative explanations for a mismatch between the number of sequences retrieved and the amount of biomass consumed are possible since they could be related to: (1) the length of the target DNA sequence (2) the affinity of the g/h primer pair for certain DNA sequences, or (3) how rich in chloroplast DNA that particular plant part was. Still, in our study we did not find a bias in detection based on sequence size. The affinity of the g/h primer pair for certain groups has been investigated *in silico* and tends to show good representation across most taxa [22]. Concordantly, we found that the proportion of DNA sequence reads was significantly correlated with the amount weighed macroscopically, which indicates that the amount of biomass consumed influences how many DNA sequences are retrieved after barcoding. Such a result is important to note, especially for species, such as wild free-ranging ungulates with mixed diets for which an experimental test would be impractical. We also note that we found a tighter correlation compared to stable isotope analyses [26] which may be explained by the different diets investigated or the methods used.

Apart from the advantage of being more sensitive and taxonomically more resolute, the DNA-based method was, in our case, also a cheaper method than the macroscopic method. The efficient time to produce data on rumen content takes 1–2 hours per rumen using the DNA-based method, from DNA extraction to a prepared data for analysis, This is 4–8 times shorter time than the macroscopic analysis, that takes about 8 hours per rumen for a trained examiner. The material costs for the DNA-based method was about €40, while materials to conduct macroscopic analyses did cost less than €1 per sample. The difference in total cost per sample thus depends largely on the expenses in salary, which in our case meant that the DNA-based method costed about €115 per sample, which was lower than the total cost of €300 per sample using the macroscopic method.

## Conclusion

By comparing two methods for dietary analysis of rumen content in free-ranging ungulates, we have seen that the classical macroscopic analysis method is not completely replaced by the newer DNA-based method. Some taxa that were identified macroscopically were not found with the DNA. It may be that such taxa were misidentified via macroscopic methods, however even some of the easily identified taxa were not identified via DNA. Nonetheless, DNA did prove to be more sensitive and taxonomically more resolute as it identified more taxa than the macroscopic methods. Moreover it showed a positive relationship to the amount of biomass identified macroscopically, suggesting that the number of DNA sequences obtained may be used as an indicator for how much biomass was consumed. DNA metabarcoding can be a powerful new method to study the diets of animals, yet we suggest that more studies be done to investigate the comparability of past and present methods using both experimental and comparative approaches.

## Supporting Information

S1 Table Rumen content of roe deer and fallow deer on family level. Number of rumens containing identified families using from one or both detection methods, i.e. DNA metabarcoding (DNA) and macroscopic identification (Macro).
(DOCX)

S2 Table Rumen content of roe deer and fallow deer on genus level. Number of rumens containing identified genera using from one or both detection methods, i.e. DNA metabarcoding (DNA) and macroscopic identification (Macro).
(DOCX)

S3 Table Rumen content of roe deer and fallow deer on species level. Number of rumens containing identified species using from one or both detection methods, i.e. DNA metabarcoding (DNA) and macroscopic identification (Macro).
(DOCX)
