# Supplementary material for: Diet Assessment Based on Rumen Contents: A Comparison between DNA Metabarcoding and Macroscopy
Source: PLoS One. 2016 Jun 20;11(6):e0157977. doi: 10.1371/journal.pone.0157977 (PMC4913902; doi:10.1371/journal.pone.0157977)
Supplement: S1 Table — Number of rumens containing identified families using from one or both detection methods, i.e. DNA metabarcoding (DNA) and macroscopic identification (Macro). (DOCX) [file pone.0157977.s001.docx]

**S1 Table.** **Rumen content of roe deer and fallow deer on family level.** Number of rumens containing identified families using from one or both detection methods, i.e. DNA-metabarcoding (DNA) and macroscopic identification (Macro).

|  | Fallow deer | | | | Roe deer | | | |
| --- | --- | --- | --- | --- | --- | --- | --- | --- |
| Family | **Both** | **Macro** | **DNA** | **Total** | **Both** | **Macro** | **DNA** | **Total** |
| Aceraceae | 1 |  | 1 | 2 |  |  | 1 | 1 |
| Apiaceae |  |  | 1 | 1 |  |  | 3 | 3 |
| Asteraceae | 2 | 1 | 6 | 9 | 2 | 1 | 2 | 5 |
| Betulaceae | 5 |  | 5 | 10 |  |  | 8 | 8 |
| Brassicaceae | 1 |  | 2 | 3 |  |  | 1 | 1 |
| Caprifoliaceae |  |  |  | 0 |  |  | 1 | 1 |
| Caryophyllaceae | 1 | 1 | 4 | 6 |  |  |  | 0 |
| Chenopodiaceae | 1 | 1 | 2 | 4 |  |  | 1 | 1 |
| Corylaceae |  |  |  | 0 |  |  | 1 | 1 |
| Cyperaceae |  | 2 | 1 | 3 |  |  |  | 0 |
| Empetraceae |  |  | 1 | 1 |  |  |  | 0 |
| Ericaceae | 9 |  |  | 9 | 4 | 1 | 3 | 8 |
| Fabaceae | 7 | 2 | 1 | 10 | 5 |  | 2 | 7 |
| Fagaceae | 4 | 1 | 1 | 6 | 4 | 2 | 1 | 7 |
| Grossulariaceae |  |  |  | 0 |  | 3 |  | 3 |
| Hypericaceae |  | 1 |  | 1 |  |  | 3 | 3 |
| Juncaceae | 3 |  | 3 | 6 |  |  |  | 0 |
| Lamiaceae |  |  | 1 | 1 |  |  |  | 0 |
| Lythraceae |  |  |  | 0 |  |  | 1 | 1 |
| Nartheciaceae |  |  | 1 | 1 |  |  |  | 0 |
| Oleaceae |  |  | 2 | 2 |  |  |  | 0 |
| Onagraceae |  |  | 1 | 1 |  |  | 3 | 3 |
| Oxalidaceae |  | 1 | 4 | 5 |  |  | 3 | 3 |
| Pinaceae | 1 | 2 | 3 | 6 |  | 3 |  | 3 |
| Plantaginaceae | 1 | 1 | 2 | 4 |  |  | 1 | 1 |
| Poaceae | 10 |  |  | 10 | 6 | 1 |  | 7 |
| Polygonaceae | 2 | 1 | 7 | 10 | 1 | 1 | 5 | 7 |
| Polypodiaceae | 1 |  | 1 | 2 | 3 |  | 1 | 4 |
| Primulaceae |  |  | 4 | 4 |  |  | 4 | 4 |
| Ranunculaceae | 3 | 1 | 6 | 10 | 3 | 1 | 4 | 8 |
| Rhamnaceae |  |  | 1 | 1 |  |  |  | 0 |
| Rosaceae | 4 |  | 5 | 9 | 8 |  | 1 | 9 |
| Salicaceae | 3 |  | 4 | 7 | 2 |  | 3 | 5 |
| Scrophulariaceae |  | 1 | 7 | 8 |  |  | 2 | 2 |
| Urticaceae |  | 1 |  | 1 |  |  |  | 0 |
| Valerianaceae |  |  |  | 0 |  |  | 2 | 2 |
| Violaceae |  |  | 1 | 1 |  |  | 2 | 2 |
| Sum | 59 | 17 | 78 | 154 | 38 | 13 | 59 | 110 |
