# Supplementary material for: Diet Assessment Based on Rumen Contents: A Comparison between DNA Metabarcoding and Macroscopy
Source: PLoS One. 2016 Jun 20;11(6):e0157977. doi: 10.1371/journal.pone.0157977 (PMC4913902; doi:10.1371/journal.pone.0157977)
Supplement: S2 Table — Number of rumens containing identified genera using from one or both detection methods, i.e. DNA metabarcoding (DNA) and macroscopic identification (Macro). (DOCX) [file pone.0157977.s002.docx]

**S2 Table.** **Rumen content of roe deer and fallow deer on genus level.** Number of rumens containing identified genera using from one or both detection methods, i.e. DNA-metabarcoding (DNA) and macroscopic identification (Macro).

|  | Fallow deer | | | | Roe deer | | | |
| --- | --- | --- | --- | --- | --- | --- | --- | --- |
| Genus | Both | Macro | DNA | Total | Both | Macro | DNA | Total |
| Acer | 1 |  | 1 | 2 |  |  | 1 | 1 |
| Achillea | 1 |  | 1 | 2 |  |  |  | 0 |
| Alnus |  |  | 5 | 5 |  |  | 1 | 1 |
| Angelica |  |  |  | 0 |  |  | 2 | 2 |
| Anthoxanthum |  |  | 1 | 1 |  |  |  | 0 |
| Anthriscus |  |  | 1 | 1 |  |  | 3 | 3 |
| Athyrium |  |  | 2 | 2 |  |  | 4 | 4 |
| Avena |  | 6 |  | 6 |  | 1 |  | 1 |
| Betula | 5 |  | 5 | 10 |  |  | 8 | 8 |
| Brassica | 1 |  | 2 | 3 |  |  | 1 | 1 |
| Calluna | 6 |  | 3 | 9 | 3 |  | 4 | 7 |
| Carex |  | 2 | 1 | 3 |  |  |  | 0 |
| Cerastium |  |  | 3 | 3 |  |  |  | 0 |
| Chenopodium | 1 | 1 | 2 | 4 |  |  | 1 | 1 |
| Corylus |  |  |  | 0 |  |  | 1 | 1 |
| Dactylis |  |  | 2 | 2 |  |  |  | 0 |
| Deschampsia | 5 | 2 | 3 | 10 |  | 4 |  | 4 |
| Elymus |  |  | 1 | 1 |  |  |  | 0 |
| Empetrum |  |  | 1 | 1 |  |  |  | 0 |
| Epilobium |  |  | 1 | 1 |  |  | 3 | 3 |
| Euphrasia |  |  | 1 | 1 |  |  |  | 0 |
| Fallopia |  |  | 1 | 1 |  |  |  | 0 |
| Festuca |  |  | 5 | 5 |  |  | 1 | 1 |
| Filipendula |  |  | 2 | 2 |  |  | 4 | 4 |
| Frangula |  |  | 1 | 1 |  |  |  | 0 |
| Fraxinus |  |  | 2 | 2 |  |  |  | 0 |
| Glyceria |  |  | 1 | 1 |  |  |  | 0 |
| Gnaphalium |  |  | 1 | 1 |  |  |  | 0 |
| Gymnocarpium |  | 1 |  | 1 |  | 2 |  | 2 |
| Holcus |  |  | 4 | 4 |  |  |  | 0 |
| Hypericum |  | 1 |  | 1 |  |  | 3 | 3 |
| Juncus | 1 | 2 | 2 | 5 |  |  |  | 0 |
| Lathyrus | 1 |  | 3 | 4 |  |  | 1 | 1 |
| Leontodon |  |  | 4 | 4 |  |  |  | 0 |
| Lotus |  |  | 6 | 6 |  |  | 1 | 1 |
| Luzula |  |  | 4 | 4 |  |  |  | 0 |
| Lysimachia |  |  | 1 | 1 |  |  | 4 | 4 |
| Lythrum |  |  |  | 0 |  |  | 1 | 1 |
| Matricaria |  | 2 |  | 2 |  |  |  | 0 |
| Melampyrum |  | 1 | 5 | 6 |  |  | 2 | 2 |
| Molinia |  |  | 1 | 1 |  |  |  | 0 |
| Narthecium |  |  | 1 | 1 |  |  |  | 0 |
| Oxalis |  | 1 | 4 | 5 |  |  | 3 | 3 |
| Persicaria |  | 1 | 2 | 3 | 1 | 1 | 1 | 3 |
| Phalaris |  |  |  | 0 |  |  | 1 | 1 |
| Picea |  | 2 | 3 | 5 |  | 1 |  | 1 |
| Pinus |  | 1 | 1 | 2 |  | 2 |  | 2 |
| Plantago | 1 | 1 | 2 | 4 |  |  | 1 | 1 |
| Polygonum |  |  | 5 | 5 |  |  |  | 0 |
| Polypodium |  |  |  | 0 |  | 1 |  | 1 |
| Populus | 1 | 1 | 1 | 3 | 2 |  | 1 | 3 |
| Potentilla |  |  | 5 | 5 |  |  | 1 | 1 |
| Prunella |  |  | 1 | 1 |  |  |  | 0 |
| Prunus |  |  | 1 | 1 |  |  |  | 0 |
| Pyrola |  |  |  | 0 |  | 1 |  | 1 |
| Quercus | 4 | 1 | 1 | 6 | 4 | 2 | 1 | 7 |
| Ranunculus | 3 | 1 | 6 | 10 | 3 | 1 | 4 | 8 |
| Ribes |  |  |  | 0 |  | 3 |  | 3 |
| Rubus | 3 |  | 6 | 9 | 8 |  | 1 | 9 |
| Rumex | 2 |  | 6 | 8 |  | 1 | 5 | 6 |
| Salix | 2 |  | 7 | 9 |  |  | 8 | 8 |
| Sambucus |  |  |  | 0 |  |  | 1 | 1 |
| Scirpus |  |  | 1 | 1 |  |  |  | 0 |
| Sorbus |  | 2 |  | 2 |  | 2 |  | 2 |
| Spergula |  |  | 1 | 1 |  |  |  | 0 |
| Spiraea |  |  | 1 | 1 |  |  |  | 0 |
| Stellaria | 1 | 1 | 2 | 4 |  |  |  | 0 |
| Taraxacum |  | 3 |  | 3 |  | 3 |  | 3 |
| Trientalis |  |  | 3 | 3 |  |  |  | 0 |
| Trifolium | 6 | 3 | 1 | 10 | 5 |  | 2 | 7 |
| Triticum | 3 | 1 | 1 | 5 | 1 |  | 2 | 3 |
| Urtica |  | 1 |  | 1 |  |  |  | 0 |
| Vaccinium | 8 |  |  | 8 | 4 |  | 2 | 6 |
| Valeriana |  |  |  | 0 |  |  | 2 | 2 |
| Veronica |  |  | 3 | 3 |  |  |  | 0 |
| Vicia |  | 1 | 2 | 3 |  |  |  | 0 |
| Viola |  |  | 1 | 1 |  |  | 2 | 2 |
| Sum | 56 | 39 | 147 | 242 | 31 | 25 | 84 | 140 |
