# Supplementary material for: Diet Assessment Based on Rumen Contents: A Comparison between DNA Metabarcoding and Macroscopy
Source: PLoS One. 2016 Jun 20;11(6):e0157977. doi: 10.1371/journal.pone.0157977 (PMC4913902; doi:10.1371/journal.pone.0157977)
Supplement: S3 Table — Number of rumens containing identified species using from one or both detection methods, i.e. DNA metabarcoding (DNA) and macroscopic identification (Macro). (DOCX) [file pone.0157977.s003.docx]

**S3 Table.** **Rumen content of roe deer and fallow deer on species level.** Number of rumens containing identified species using from one or both detection methods, i.e. DNA-metabarcoding (DNA) and macroscopic identification (Macro).

|  | Fallow deer | | | | Roe deer | | | |
| --- | --- | --- | --- | --- | --- | --- | --- | --- |
| Species | Both | Macro | DNA | Total | Both | Macro | DNA | Total |
| Acer platanoides |  | 1 |  | 1 |  |  |  | 0 |
| Achillea millefolium |  | 1 |  | 1 |  |  |  | 0 |
| Achillea ptarmica |  | 1 |  | 1 |  |  |  | 0 |
| Angelica sylvestris |  |  |  | 0 |  |  | 2 | 2 |
| Anthoxanthum odoratum |  |  | 1 | 1 |  |  |  | 0 |
| Anthriscus sylvestris |  |  | 1 | 1 |  |  | 3 | 3 |
| Avena sativa |  | 6 |  | 6 |  | 1 |  | 1 |
| Brassica napus |  |  | 3 | 3 |  |  | 1 | 1 |
| Calluna vulgaris | 6 |  | 3 | 9 | 3 |  | 4 | 7 |
| Carex pallescens |  |  | 1 | 1 |  |  |  | 0 |
| Cerastium fontanum |  |  | 3 | 3 |  |  |  | 0 |
| Corylus avellana |  |  |  | 0 |  |  | 1 | 1 |
| Dactylis glomerata |  |  | 2 | 2 |  |  |  | 0 |
| Deschampsia cespitosa |  |  | 4 | 4 |  |  |  | 0 |
| Deschampsia flexuosa | 4 | 3 | 2 | 9 |  | 4 |  | 4 |
| Elymus repens |  |  | 1 | 1 |  |  |  | 0 |
| Empetrum nigrum |  |  | 1 | 1 |  |  |  | 0 |
| Epilobium angustifolium |  |  |  | 0 |  |  | 2 | 2 |
| Epilobium palustre |  |  | 1 | 1 |  |  | 3 | 3 |
| Fallopia convolvulus |  |  | 1 | 1 |  |  |  | 0 |
| Festuca pratensis |  |  | 5 | 5 |  |  | 1 | 1 |
| Filipendula ulmaria |  |  | 2 | 2 |  |  | 4 | 4 |
| Frangula alnus |  |  | 1 | 1 |  |  |  | 0 |
| Fraxinus excelsior |  |  | 2 | 2 |  |  |  | 0 |
| Gnaphalium uliginosum |  |  | 1 | 1 |  |  |  | 0 |
| Gymnocarpium dryopteris |  | 1 |  | 1 |  | 2 |  | 2 |
| Hypericum maculatum |  | 1 |  | 1 |  |  | 3 | 3 |
| Lathyrus pratensis |  | 1 | 3 | 4 |  |  | 1 | 1 |
| Leontodon autumnalis |  |  | 4 | 4 |  |  |  | 0 |
| Luzula atlantica |  |  | 1 | 1 |  |  |  | 0 |
| Luzula pilosa |  |  | 4 | 4 |  |  |  | 0 |
| Lysimachia vulgaris |  |  | 1 | 1 |  |  | 4 | 4 |
| Matricaria perforata |  | 2 |  | 2 |  |  |  | 0 |
| Melampyrum pratense |  |  | 5 | 5 |  |  | 2 | 2 |
| Molinia caerulea |  |  | 1 | 1 |  |  |  | 0 |
| Narthecium ossifragum |  |  | 1 | 1 |  |  |  | 0 |
| Oxalis acetosella |  | 1 | 4 | 5 |  |  | 3 | 3 |
| Persicaria lapathifolia |  | 1 |  | 1 |  | 2 |  | 2 |
| Phalaris arundinacea |  |  |  | 0 |  |  | 1 | 1 |
| Picea abies |  | 2 |  | 2 |  | 1 |  | 1 |
| Pinus sylvestris |  | 1 | 1 | 2 |  | 2 |  | 2 |
| Plantago lanceolata |  |  | 1 | 1 |  |  | 1 | 1 |
| Plantago major | 1 | 1 | 2 | 4 |  |  |  | 0 |
| Polygonum aviculare |  |  | 5 | 5 |  |  |  | 0 |
| Polypodium vulgare |  |  |  | 0 |  | 1 |  | 1 |
| Populus tremula | 1 |  | 1 | 2 | 2 |  | 1 | 3 |
| Potentilla erecta |  |  | 5 | 5 |  |  | 1 | 1 |
| Prunella vulgaris |  |  | 1 | 1 |  |  |  | 0 |
| Prunus padus |  |  | 1 | 1 |  |  |  | 0 |
| Quercus robur | 4 | 1 | 1 | 6 | 4 | 2 | 1 | 7 |
| Ranunculus repens |  | 1 |  | 1 |  |  |  | 0 |
| Rubus chamaemorus |  |  |  | 0 |  |  | 3 | 3 |
| Rubus idaeus | 3 |  | 6 | 9 | 6 | 2 |  | 8 |
| Rumex acetosella |  | 1 |  | 1 |  |  |  | 0 |
| Salix triandra |  |  | 1 | 1 |  |  |  | 0 |
| Sambucus nigra |  |  |  | 0 |  |  | 1 | 1 |
| Sorbus aucuparia |  | 2 |  | 2 |  | 2 |  | 2 |
| Spiraea salicifolia |  |  | 1 | 1 |  |  |  | 0 |
| Stellaria graminea |  | 1 |  | 1 |  |  |  | 0 |
| Trientalis europaea |  |  | 3 | 3 |  |  |  | 0 |
| Trifolium medium |  | 1 |  | 1 |  |  |  | 0 |
| Trifolium repens |  | 1 |  | 1 |  |  |  | 0 |
| Triticum aestivum | 3 | 1 | 1 | 5 | 1 |  | 2 | 3 |
| Urtica dioica |  | 5 |  | 5 |  |  |  | 0 |
| Vaccinium myrtillus | 5 |  | 2 | 7 | 4 |  | 2 | 6 |
| Vaccinium uliginosum | 2 |  | 5 | 7 |  |  | 4 | 4 |
| Vaccinium vitis-idaea | 3 |  | 4 | 7 | 1 | 1 | 2 | 4 |
| Veronica chamaedrys |  |  | 3 | 3 |  |  |  | 0 |
| Vicia cracca |  |  | 2 | 2 |  |  |  | 0 |
| Sum | 32 | 37 | 105 | 174 | 21 | 20 | 53 | 94 |
